# ANPEP governs macrophage lipid metabolism and macrophage foam cell formation in large-artery atherosclerotic stroke

**DOI:** 10.3389/fimmu.2026.1747321

**Published:** 2026-04-24

**Authors:** Fanghui Bai, Yuanzheng Hu, Zongqing Wang, Yue Guo, Pengkun Bie, Zhanwang Shi, Xiangxin Li, DanDan Li, Chengpeng Zhao, Qian Xu

**Affiliations:** 1 Department of Neurology, Nanyang Central Hospital, Nanyang, China; 2 Henan Provincial Engineering Laboratory of Insects Bio-Reactor, Nanyang Normal University, Nanyang, China

**Keywords:** ANPEP, large-artery atherosclerotic stroke, lipid metabolism, lysosome, macrophage foam cell formation

## Abstract

**Background:**

Large-artery (LAA-stroke) is a leading cause of cardiovascular morbidity and mortality worldwide. Recent evidence indicates that lysosomal dysfunction plays a critical role in its pathogenesis, yet the underlying molecular mechanisms remain incompletely understood. Identifying characteristic lysosome-related genes (LRGs) offers new perspectives for deciphering disease mechanisms and developing novel therapeutic strategies.

**Methods:**

This study integrated multi-omics data and experimental validation to identify key LRGs in LAA-stroke. We analyzed transcriptomic datasets (GSE100927 and GSE28829) through differential expression analysis, protein-protein interaction(PPI) network construction, and machine learning algorithms to screen characteristic LRGs. Single-cell RNA sequencing data (GSE159677) were utilized to resolve gene expression patterns across cellular subpopulations in atherosclerotic plaques. A series of experimental approaches, including in silico knockout, RNA sequencing, and *in vitro* assays, were employed to validate the role of ANPEP in macrophage foam cell formation.

**Results:**

We identified seven characteristic genes from 63 differentially expressed LRGs. Among them, Aminopeptidase N(ANPEP) demonstrated outstanding diagnostic performance in both training and external validation cohorts (AUC: 0.803–1.000). Single-cell analysis revealed specific enrichment of ANPEP in macrophages, particularly in the TREM2+ lipid-associated macrophage subset. Pseudotime trajectory analysis indicated that ANPEP expression closely aligned with lipid transport genes such as SPP1, CD36, and ABCA1. Functional experiments confirmed that ANPEP knockdown significantly suppressed ox-LDL-induced foam cell formation and intracellular lipid deposition by modulating lipid metabolism pathways, including VDR and MAPK signaling.

**Conclusions:**

This multi-level study suggests that ANPEP may serve as a key regulator of macrophage lipid metabolism in LAA-stroke. Our findings provide evidence supporting its potential as a novel candidate biomarker and highlight its promise as a therapeutic target for modulating lipid metabolic disorders in cardiovascular diseases.

## 1 Introduction

Atherosclerosis (AS) is a progressive, multifactorial chronic disease characterized by abnormal cholesterol and lipid accumulation in the arterial wall, leading to atherosclerotic plaque formation. Among its complications, LAA-stroke represents one of the most severe acute manifestations, primarily resulting from vulnerable plaque rupture in intracranial or carotid arteries, which triggers secondary thrombosis and acute vascular occlusion (1). As a major contributor to global morbidity and mortality, LAA-stroke reflects both the endpoint of AS and the pathological progression of plaques from stable to inflammatory, structurally vulnerable states (2). Thus, elucidating the molecular mechanisms of plaque progression and acute ischemic events is clinically crucial for developing targeted preventive and therapeutic strategies.

AS pathogenesis involves multiple cell types and biological mechanisms, with macrophages playing a pivotal role. Macrophages form atherosclerotic plaques by internalizing oxidized low-density lipoprotein (ox-LDL) and transforming into foam cells, a key step in AS pathogenesis (3). Excessive ox-LDL uptake via scavenger receptors induces abnormal cholesterol ester accumulation in macrophages, triggering their transformation into foam cells. These cells not only form the plaque lipid core but also exacerbate local inflammation and fibrous cap degradation by secreting pro-inflammatory cytokines and matrix metalloproteinases, promoting plaque destabilization (4). Identifying key molecules regulating macrophage cholesterol metabolism and phenotypic transformation is therefore critical for understanding LAA-stroke pathogenesis. Recent studies indicate that lysosomal functional integrity in macrophages maintains cholesterol metabolic homeostasis. Lysosomes act as hubs for intracellular lipid degradation and cholesterol efflux, and regulate inflammasome activation, autophagy flux, and apoptosis (5). Increased lysosomal membrane permeability or abnormal hydrolase activity induces cholesterol crystal deposition, amplified inflammation, and cell death, directly driving atherosclerotic plaque progression and associated complications (6). Macrophage lysosomes participate in both the initiation and progression of atherosclerotic plaques (7). Recent research highlights the critical role of lysosomal function and associated genes in AS pathogenesis (6). Lysosomes are integral to intracellular lipid metabolism and critical for autophagy and cellular clearance. However, specific lysosome-associated genes critical for macrophage foam cell formation and LAA-stroke progression remain to be systematically identified and mechanistically validated.

Aminopeptidase N (ANPEP), also known as CD13, is a zinc-dependent metalloprotease and a recently recognized lysosome-associated functional molecule (8). Widely expressed on monocytes/macrophages, endothelial cells, and smooth muscle cells, ANPEP participates in peptide hormone activation, antigen presentation, and inflammatory regulation by hydrolyzing N-terminal amino acids from peptide chains (9). Notably, ANPEP is not restricted to the cell membrane; after internalization, it traffics to lysosomes, participating in intracellular protein turnover and autophagy regulation (10). Existing studies show ANPEP regulates macrophage activation and phagocytosis in inflammatory diseases and the tumor microenvironment (11). However, its expression patterns, cell-specific distribution in LAA-stroke, and causal relationship with foam cell formation remain unknown. Given ANPEP’s role in linking lysosomal function and inflammation, we hypothesize it participates in LAA-stroke pathological progression by regulating macrophage lysosomal homeostasis, thereby influencing cholesterol metabolism and inflammatory phenotypes.

This study aimed to identify differentially expressed genes (DEGs) using public datasets, screen potential lysosome-associated signature genes for LAA-stroke via machine learning, and validate these genes in clinical samples. Cells associated with these signature genes were identified in single-cell datasets and validated via *in vitro* experiments. We then knocked down the signature gene ANPEP to explore the relationship between lysosomal genes and macrophage foam cell progression, thereby providing new insights into the pathogenesis of large artery atherosclerotic stroke.

## 2 Materials and methods

### 2.1 Data source

In this study, we acquired gene expression microarray data from the Gene Expression Omnibus (GEO) database (http://www.ncbi.nlm.nih.gov/geo) (12). Specifically, data were sourced from the GPL17077 platform with accession number GSE100927 (13) (comprising 69 samples from the AS group and 35 from the control group), the GPL570 platform with accession number GSE28829 (14) (including 16 samples from advanced atherosclerotic plaques and 13 from early atherosclerotic plaques), and the GPL18573 platform with accession number GSE159677 (15). The GSE100927 dataset, with its relatively larger sample size, provided stability for model construction and represented the transcriptional profile of a general AS population, and was thus used as the training set. The GSE28829 dataset, encompassing different disease stages (early vs. advanced plaques), was utilized as an external validation set to enhance the generalizability and clinical relevance of our results. The GSE159677 dataset was utilized for single-cell RNA sequencing (scRNA-seq) analysis. We obtained 883 LRGs from the gene set enrichment analysis (GSEA) database (16).

### 2.2 Identification of DEGs

The DEGs from dataset GSE100927 were identified utilizing the Limma R package applied to normalized count data (17). DEGs were selected based on an absolute Log2 fold change greater than 1 and an adjusted P-value less than 0.05. Visual representations of the DEGs, including a heatmap and a volcano plot, were generated using the pheatmap and ggplot2 R packages, respectively.

### 2.3 Enrichment analyses

To elucidate the potential biological functions and underlying mechanisms of the identified genes, we employed the R package “clusterProfiler” (18) to conduct an enrichment analysis of Gene Ontology (GO) (19) terms and Kyoto Encyclopedia of Genes and Genomes (KEGG) (20) pathways for the target genes. GO terms, encompassing biological processes (BP), cellular components (CC), and molecular functions (MF), along with KEGG pathways, were deemed statistically significant if they exhibited an adjusted P-value of less than 0.05. To conduct functional annotation of DEGs through GO and the KEGG, we employed Metascape (http://metascape.org), a widely utilized online resource in biological research. The DEGs were further analyzed for PPIs using version 10.5 of the STRING database (http://www.string-db.org/) (21), which computes a combined score to evaluate the reliability of interactions, with a threshold of greater than 0.4 deemed significant.

### 2.4 Screening of characteristic genes

Differentially expressed lysosome-related genes (DELRGs) were screened to identify characteristic LRGs using two machine learning algorithms: the Least Absolute Shrinkage and Selection Operator (LASSO) and support vector machine-recursive feature elimination (SVM-RFE) (22). For LASSO regression analysis, the glmnet R package was applied, with 10-fold cross-validation adopted to optimize the penalty parameter (λ) and avoid overfitting; the optimal λ value was determined based on the minimum mean cross-validation error. For SVM-RFE analysis, classification models were constructed using the e1071, kernlab and Caret R packages, where grid search cross-validation was used for hyperparameter tuning (including cost and gamma values) to optimize model performance. Finally, the intersection of characteristic LRGs identified by the two algorithms was obtained and visualized using the Venn R package.

### 2.5 Immune infiltration analysis

The relative abundances of 22 immune cell types in the samples from GSE100927 were determined using the CIBERSORT (23, 24) algorithm. The “vioplot” package was employed to facilitate a comparative analysis of the immune cell levels between the AS and control samples.

### 2.6 Single-cell analysis

We integrated transcriptomic data from atherosclerotic core (AC) plaques and patient-matched proximal adjacent (PA) regions obtained from GSE159677. Subsequently, we performed single-cell RNA sequencing (scRNA-seq) analysis using the “Seurat”, “tidyverse” and “Matrix” R packages. The scRNA-seq data analysis included the exclusion of low-quality cells, specifically those with fewer than 200 expressed genes and those with mitochondrial content exceeding 5%, Gene expression data underwent log normalization to mitigate technical biases. Subsequently, genes exhibiting high variability (exceeding 2500) were selected for further analysis. Dimensionality reduction was conducted to enhance data visualization, employing the uniform manifold approximation and projection (UMAP) technique. Each cellular subpopulation was then annotated with a specific cell type, based on established cell type-specific marker genes (25). Cellular annotations were manually assigned based on gene expression profiles. CellChat was used to identify cell–cell communication based on a human database (26). The AUcell Score algorithm was used to scoring gene sets within scRNA-seq datasets. The monocle2 algorithm delineated the developmental trajectory (27).

### 2.7 Blood sample collection and preparation

The study comprised 12 healthy control participants and 14 patients who were consecutively recruited from Nanyang Central Hospital in Nanyang, China. The diagnostic process involved collecting detailed information on the patients’ medical history, clinical presentations, and intracranial angiography results, which were subsequently reviewed by at least two specialized neurologists. Stroke subtypes were classified according to the Trial of Org 10172 in Acute Stroke Treatment (TOAST) classification system (28) In patients diagnosed with acute ischemic stroke, confirmation was achieved via intracranial angiography and imaging techniques. Demographic data and clinical information were collected through an analysis of the patients’ medical records and a thorough investigation of their medical histories. The inclusion criteria for LAA stroke were as follows: (1) imaging indicating >50% occlusion or stenosis of the common carotid artery, anterior and posterior cerebral artery, or vertebrobasilar artery caused by atherosclerosis; (2) audible murmur detected during neck auscultation; and (3) lesion diameter >1.5 cm according to imaging results. Patients with cardioembolic stroke or stroke from other causes were excluded. Twelve volunteers, matched for age and sex, were recruited to serve as healthy controls. These participants had no documented history of neurological events, such as cerebrovascular strokes or transient ischemic attacks. Informed consent was obtained from all participants or their legal representatives prior to their inclusion in the study. The study protocol received approval from the Ethics Committee of Nanyang Central Hospital. Peripheral venous blood samples were collected from the subjects for RNA extraction and subsequent analysis.

### 2.8 Total RNA extraction and quantitative real-time PCR analysis

Upon collection, peripheral blood samples were promptly preserved in blood RNA storage tubes (Bioteke Corporation, Beijing, China).Total RNA was extracted from these preserved samples using the Eastep^®^ Super Total RNA extraction kit, with genomic DNA (gDNA) digestion included during RNA isolation. RNA purity and concentration were determined using a NanoDrop 2000 spectrophotometer (Thermo Fisher Scientific). Subsequently, cDNA synthesis was carried out using an M5 Super plus qRT–PCR RT kit (Mei5 Biotechnology Co. Ltd., Beijing, China).To quantify the expression levels of the target genes, qRT–PCR was performed using SYBR Green (TaKaRa) and the CFX96TM Real-Time System (Bio-Rad, Hercules, CA, USA).The 2^−△△^CT method was utilized for relative quantification, with GAPDH serving as the internal reference. ANPEP gene expression was determined by qRT-PCR following the method described previously. The sequences of the primers used are listed in **Supplementary Table S4**.

### 2.9 In silico macrophages perturbation

To gain mechanistic insight into the role of the top-ranked hub gene ANPEP, we performed a virtual knockout using the “scTenifoldKnk” R package on the macrophages derived from atherosclerotic plaques single-cell datasets (29).

### 2.10 Lentiviral transduction and construction of stable cell lines

To investigate the functional role of ANPEP, we established stable ANPEP-knockdown RAW264.7 cell lines via lentiviral transduction for subsequent functional studies. RAW264.7 cells were purchased from Shanghai Biotech. Cells were cultured in RPMI-1640 medium supplemented with 10% fetal bovine serum (FBS) and 1% penicillin-streptomycin solution under standard conditions. A lentivirus-based packaging system was developed using the plvx-shRNA2-ZSGreen-T2A-puro lentiviral vector (Oligobio, Beijing, China). The shRNA-ANPEP and scramble-shRNA control (sh-NC) sequences were synthesized by Oligobio (Beijing, China). At a confluence of 30%-50%, cells were transduced with lentiviral particles at a multiplicity of infection of 50 and incubated for 48 h. The medium was then replaced with fresh complete medium, and 5 µg/mL of puromycin was added to eliminate uninfected cells. Infection efficiency was assessed by fluorescence microscopy, and stable integration was validated by RT-qPCR. Specifically, total RNA was extracted from the puromycin-selected stable cell lines using the Eastep^®^ Super Total RNA extraction kit, and cDNA was synthesized with the M5 Super plus qRT–PCR RT kit (Mei5 Biotechnology Co. Ltd., Beijing, China). RT-qPCR was performed using SYBR Green (TaKaRa) and the CFX96TM Real-Time System (Bio-Rad, Hercules, CA, USA) to detect the expression level of ANPEP, with GAPDH as the internal reference; the 2−^ΔΔ^CT method was used for relative quantification to confirm stable integration of the lentiviral vector and effective knockdown of ANPEP.

### 2.11 RNA sequencing of RAW264.7 cell

Total RNA was extracted from ANPEP-knockdown RAW264.7 cells and control cells using RNA Isolator Total RNA Extraction Reagent (Vazyme, Nanjing, China, #R401-01). RNA sequencing (RNA-seq) analysis was performed by Shanghai Biotechnology Co., Ltd. (Shanghai, China). The cDNA library was sequenced using the Illumina NovaSeq platform. For gene-expression analysis, gene fragments were counted and subsequently normalized to Transcripts Per Million (TPM).DEGs were identified based on the following criteria: |log_2_(fold change)| > 1 and p-value < 0.05. GO functional categorization was performed based on molecular function, cellular component, and biological process using R software. KEGG pathway analysis was performed as previously described, with significant enrichment defined using an FDR < 0.05 as a threshold. Additionally, Eukaryotic Orthologous Groups (KOG) analysis was conducted to classify the functions of DEGs into broader functional categories (30). GSEA was also performed to identify gene sets significantly enriched in ANPEP-knockdown cells compared to control cells, providing insights into differentially regulated biological pathways and processes.

### 2.12 Cell viability (CCK-8 assay)

Oxidized low-density lipoprotein (ox-LDL, 20605ES05) was purchased from Shanghai Yeasen BioTechnologies Co., Ltd.; its endotoxin level was confirmed to be < 0.1 EU/mL. The impact of ox-LDL on cell viability was assessed using the Cell Counting Kit-8 (CCK8) assay by measuring the optical density at 450 nm (Thermo, USA). RAW264.7 cells were initially seeded into 96-well plates at a density of 1 × 10³ cells/well in 96-well plates. Upon cell adherence, the culture medium was replaced with a medium containing 3% FBS and varying concentrations of ox-LDL (0, 20, 30, 40, 50, 60 mg/mL). The cells were subsequently incubated for an additional 24 hours. Following incubation, 10 μL of CCK8 reagent (Vazyme, China) was added to each well, and the cells were cultured for an additional 2 hours at 37 °C. This procedure was conducted with three independent biological replicates.

### 2.13 Oil Red O staining

To perform Oil Red O staining, cells were first fixed with 4% paraformaldehyde. After fixation, the cells were washed with water to remove any residual fixative. The staining solution, consisting of Oil Red O dissolved in a mixture of isopropanol and water, was then applied to the cells, allowing lipid droplets to be stained red. Following incubation, excess stain was washed off with water. Finally, the stained cells were examined under a microscope to assess the presence and quantity of lipid droplets.

### 2.14 Cell immunofluorescence

The cultured cells were washed three times with PBS. After fixation with 4% polyformaldehyde for 30 min, the cells were washed three times with PBS. Next, the cells were permeabilized for 30 min with 0.5% Triton- × 100 and then washed three times with PBS. The cells were blocked with 5% BSA for 30 min and then incubated with anti-ANPEP antibody in a humidified chamber overnight at 4 °C. Cells were washed with PBS and then incubated with secondary antibody for 60 min at room temperature in the dark. After washing, the cells were incubated with DAPI solution in the dark for 5 min to stain the cell nuclei. Cell culture images were taken using a Primovert microscope (Zeiss, USA).

### 2.15 Statistical analysis

All statistical analyses in this study were performed using GraphPad Prism 8 (https://www.graphpad.com/scientific-software/prism/) and R software (http://www.R-project.org/, version 4.3.1). Unpaired Student’s t-tests were applied for continuous variables, whereas Fisher’s exact tests were used for categorical variables. A *P value* < 0.05 was regarded as statistically significant.

## 3 Results

### 3.1 Identification of DEGs

**Figure 1:**
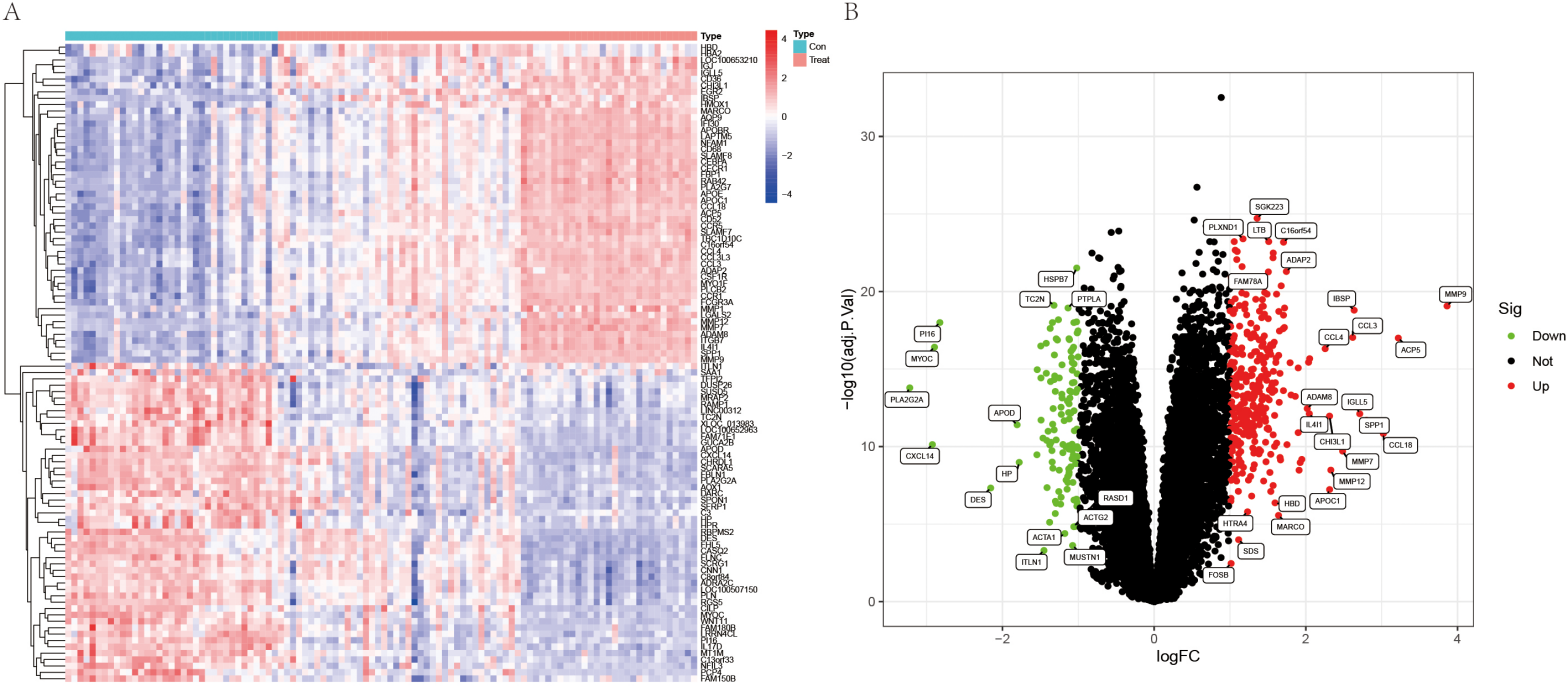
Results of differentially expressed gene analysis. Heatmaps **(A)** and volcano plots **(B)** of the differentially expressed genes (DEGs).

Differential gene expression analysis was conducted utilizing datasets GSE100927, resulting in the identification of 441 DEGs characterized by a |log_2_FC| > 1 and an adjusted p-value < 0.05 when comparing AS samples to control samples. Among these DEGs, 321 were downregulated, while 120 were upregulated (**Supplementary Table S1**). The expression patterns of these DEGs are illustrated in **Figure 1A** (heatmap) and 1B (volcano plot).

### 3.2 Enrichment function analysis of DELRGs

**Figure 2:**
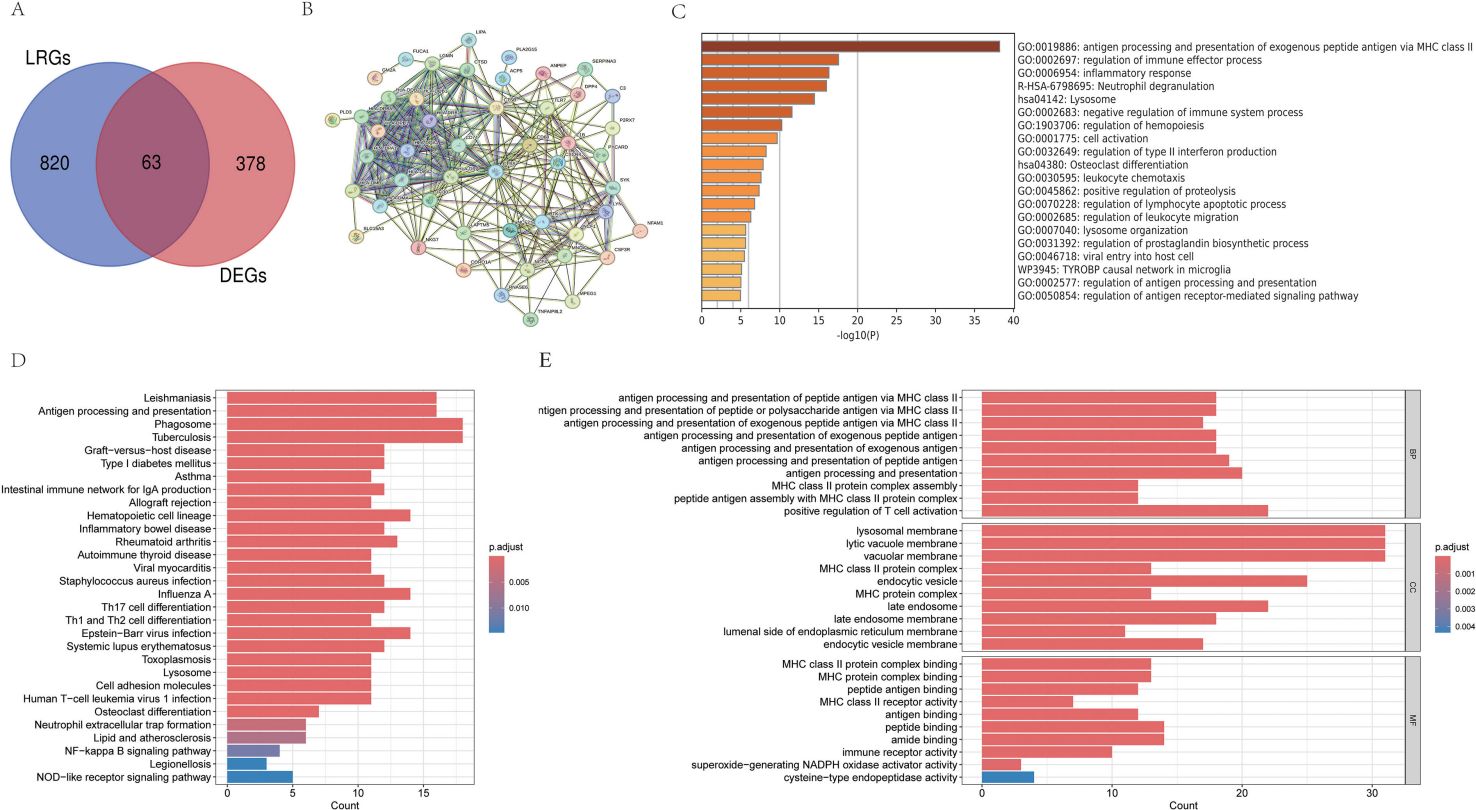
Enrichment analysis. Differential genes intersect with lysosome-related genes **(A)**. Intersection genes were used for PPI network construction **(B)**, Metascape enrichment analysis **(C)**, KEGG enrichment analysis **(D)** and GO enrichment analysis **(E)**.

A comprehensive set of 883 LRGs was sourced from the GSEA database. Subsequent intersection analysis with the identified DEGs resulted in 63 DELRGs (**Figure 2A**). To investigate the proteins associated with these 63 DELRGs, a PPI network was constructed using the STRING database (**Figure 2B**). Functional enrichment analysis indicated that the DELRGs are significantly implicated in biological processes including antigen processing and presentation of exogenous peptide antigens via MHC class II, regulation of immune effector processes, inflammatory response, neutrophil degranulation, and lysosomal function (**Figure 2C**). KEGG pathway analysis revealed that the DELRGs were predominantly enriched in pathways related to antigen processing and presentation, phagosome, lysosome, and the NF-kappa B signaling pathway (**Figure 2D**). GO analysis further demonstrated significant enrichment of DELRGs in processes such as positive regulation of T cell activation, lysosomal membrane dynamics, and peptide binding. Across BP, MF, and CC domains, the DELRGs exhibited a strong association with the major histocompatibility complex (MHC), particularly in functions such as antigen processing and presentation of peptide antigens via MHC class II, the MHC class II protein complex, the MHC protein complex, and MHC class II protein complex binding (**Figure 2E**).

### 3.3 Discerning the characteristic LRGs

**Figure 3:**
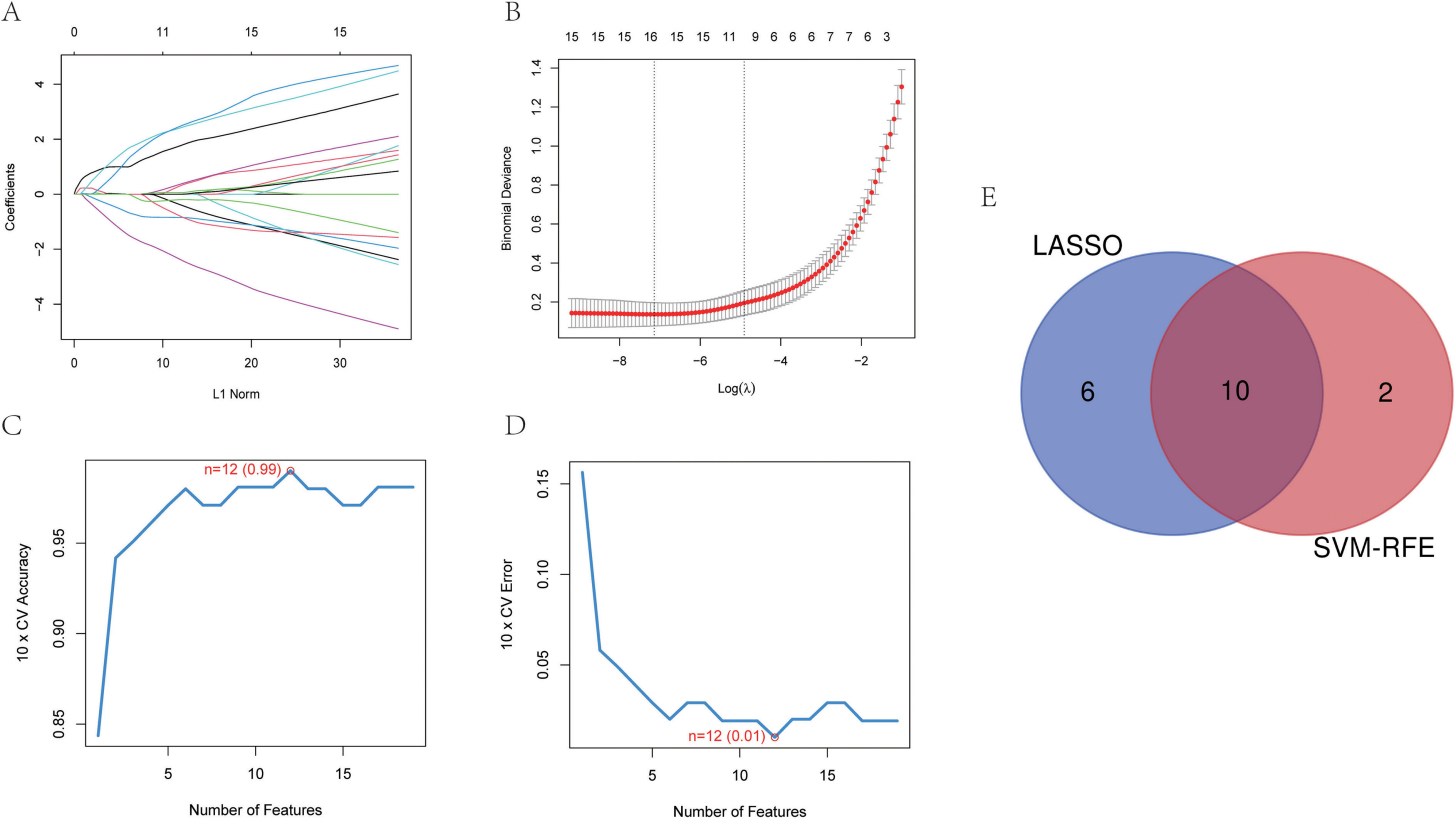
Machine learning algorithms for screening diagnostic genes. The LASSO regression algorithm **(A, B)** and SVM-RF algorithm **(C, D)** were applied to screen key gene of LAA. Venn diagram shows the intersection of diagnostic markers obtained by the two algorithms **(E)**.

**Figure 4:**
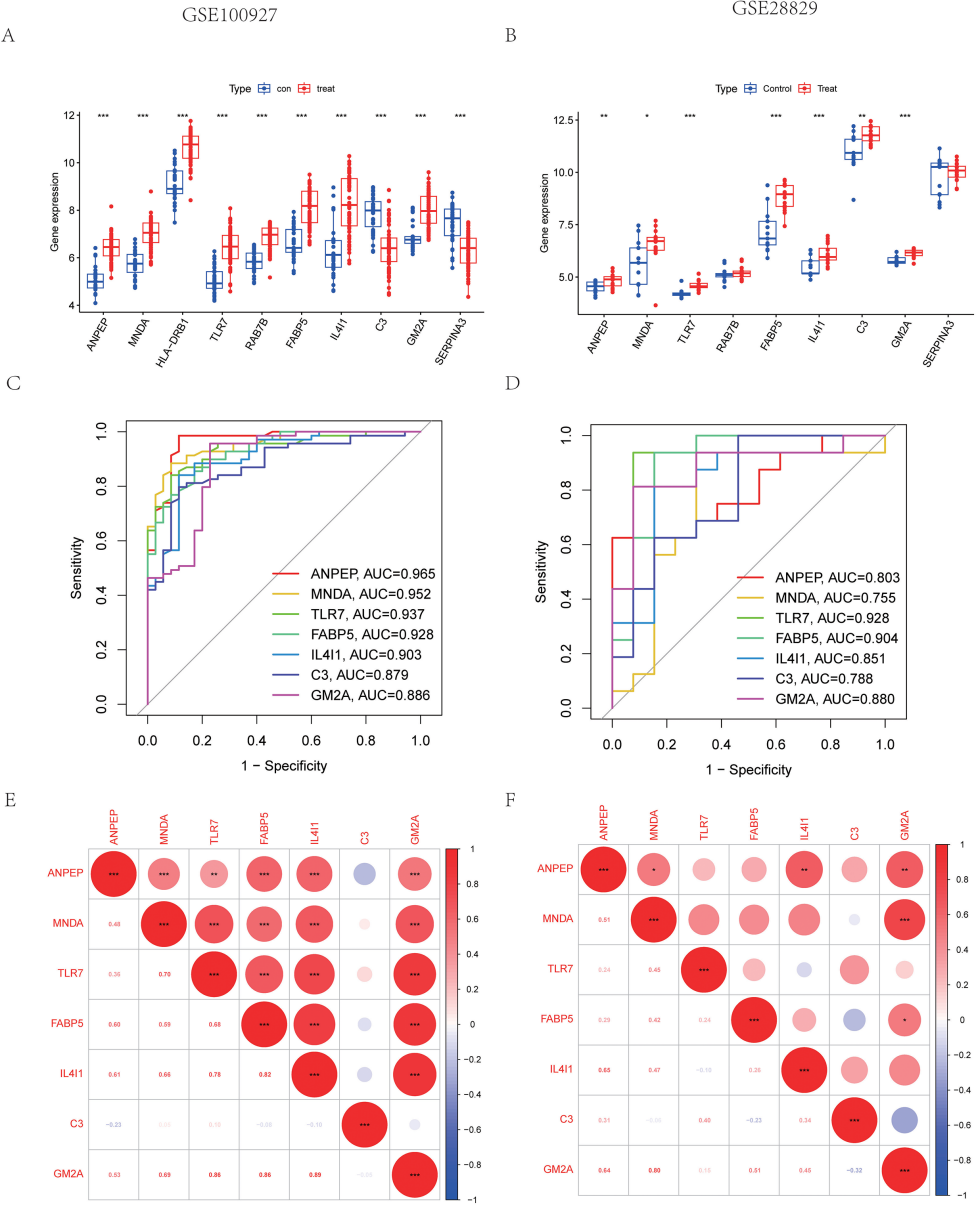
Identification of the diagnostic genes. Expression of the diagnostic genes in the training set and validation sets **(A, B)**. The ROC curve was used to evaluate the diagnostic efficiency in both the training set and the validation set **(C, D)**. Mapping the diagnostic gene correlations in the training set and validation set **(E, F)**. *P<0.05; **P<0.01; ***P<0.001.

Two machine learning approaches LASSO and SVM-RPE were employed for selecting characteristic genes among DELRGs. LASSO selected 16 characteristic genes (**Figures 3A, B**), while SVM-RFE selected 12 (**Figures 3C, D**). After intersection, ten characteristic genes were finally determined, including *TLR7, IL4I1, C3, SERPINA3, MNDA, HLA-DRB1, GM2A, ANPEP, RAB7B*, and *FABP5* (**Figure 3E**). To assess the reliability of these genes, we performed external validation using the GSE28829 datasets. Our analysis identified seven genes—*ANPEP, MNDA, TLR7, FABP5, IL4I1, C3*, and *GM2A*—as significantly differentially expressed in GSE28829 (P < 0.001, **Figure 4B**). We further examined the disease-discriminatory efficacy of these seven genes by constructing Receiver Operating Characteristic (ROC) curves using both the training set (GSE100927) and the external validation set (GSE28829). In the training cohort, the Area Under the Curve (AUC) values for *ANPEP, MNDA, TLR7, FABP5, IL4I1, C3*, and *GM2A* were 0.965, 0.952, 0.937, 0.928, 0.903, 0.879, and 0.886, respectively (**Figure 4C**). These genes also exhibited strong diagnostic performance in the external validation cohort, with AUC values of 0.803, 0.775, 0.928, 0.904, 0.851, 0.788, and 0.880, respectively (**Figure 4D**). Based on these validation outcomes, we selected these seven genes for further biological function interpretation. Furthermore, correlation analysis conducted among these candidate genes demonstrated significant intergenic relationships (**Figures 4E, F**), offering valuable insights into their potential collaborative roles in the regulation of lipid metabolism.

### 3.4 Single-cell analysis

**Figure 5:**
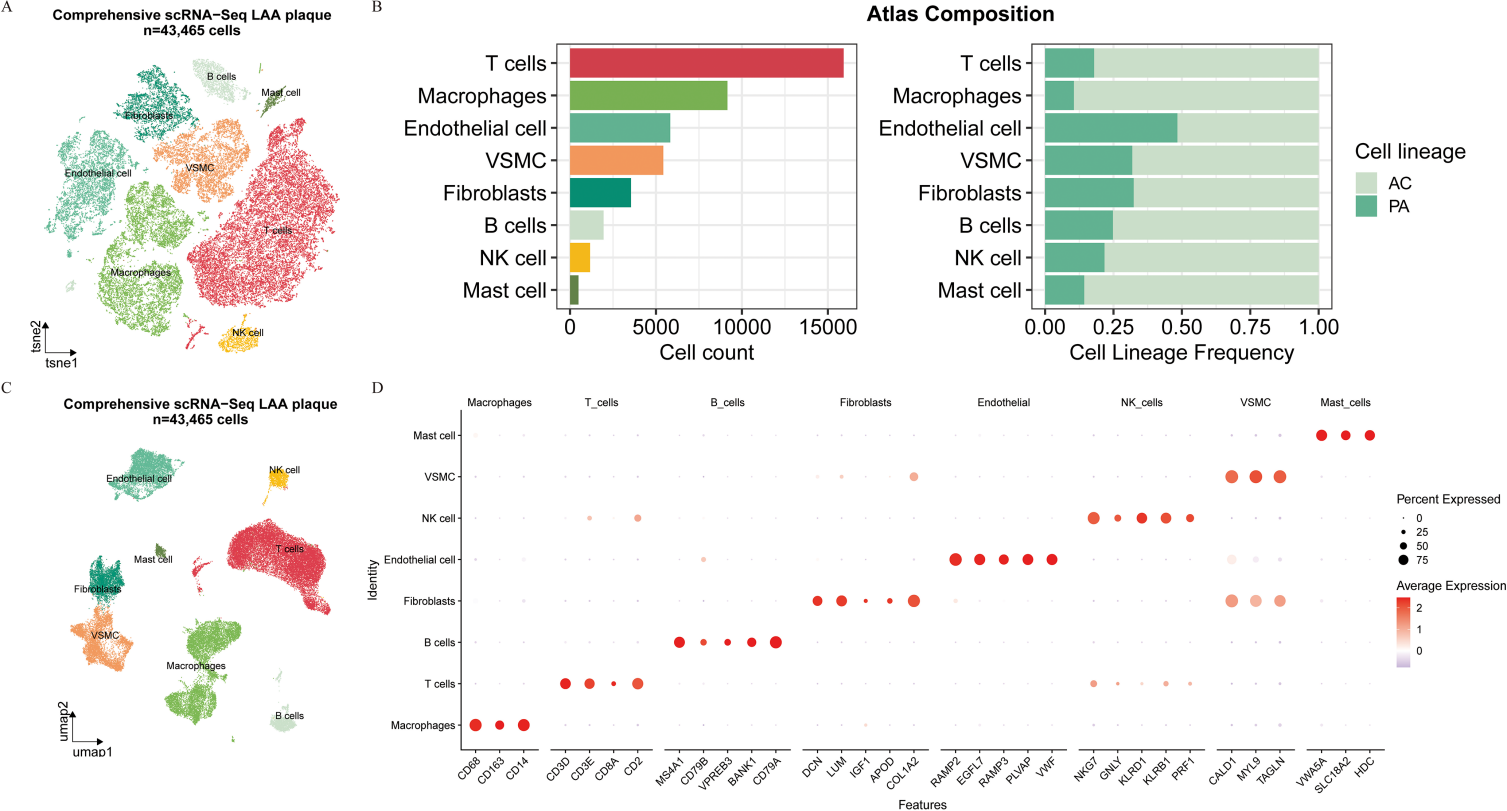
Single-cell landscape in atherosclerotic plaque. **(A)** t-SNE plot of single cells profiled in atherosclerotic plaque. **(B)** Cell proportions in each samples. **(C)** UMAP plot of single cells profiled in atherosclerotic plaque. **(D)** Bubble plot of the average and percent expression of different markers in each cell types.

**Figure 6:**
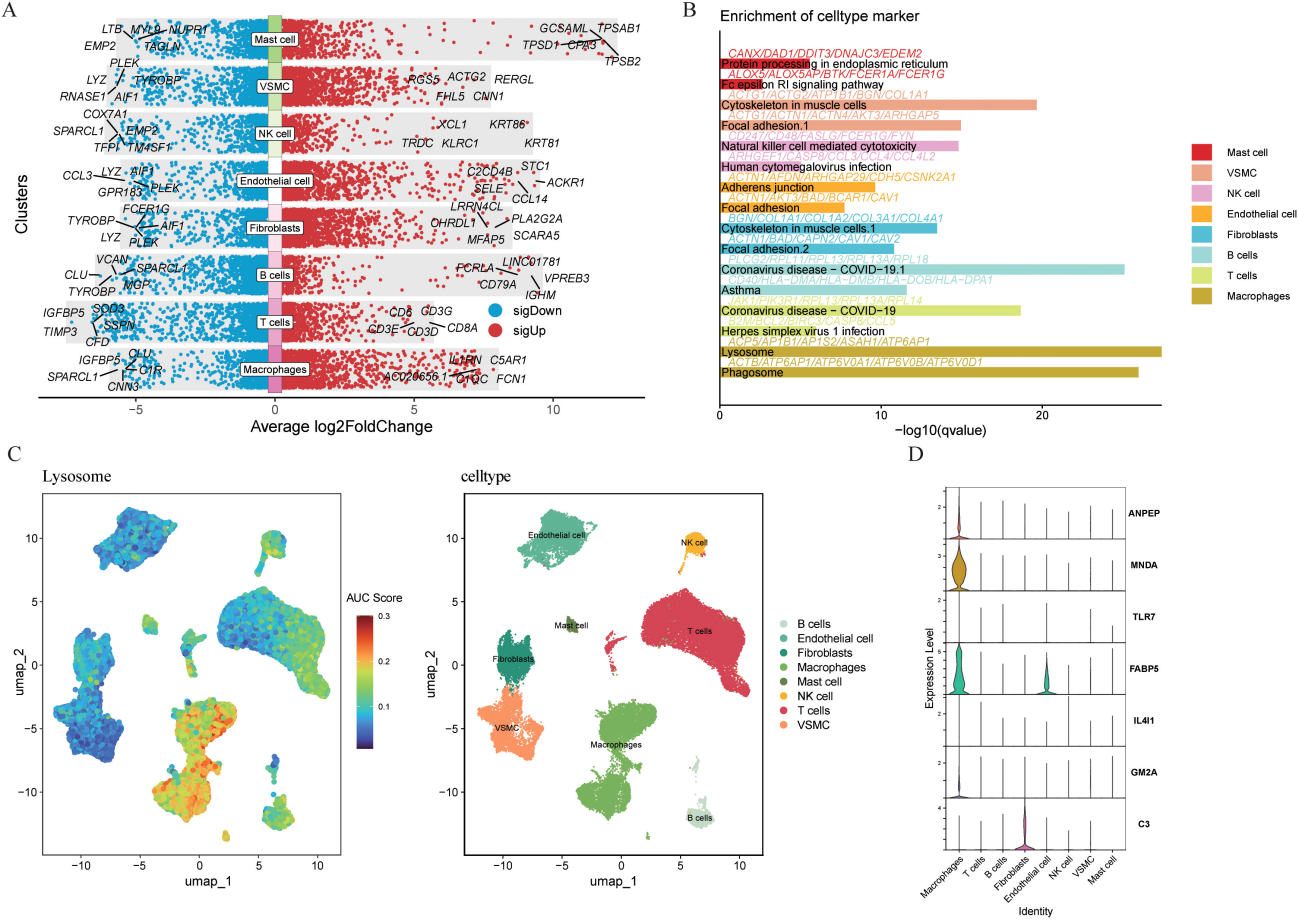
Single cell analysis. **(A)** Differential gene expression analysis reveals up and down-regulated genes across various cell types. An adjusted p value < 0.01 is indicated in red, while an adjusted p value ≥ 0.01 is indicated in black. **(B)** Enrichment of celltype marker analysis. **(C)** Visualization of lysosome-related genes activity scores across the cohort using the AUcell algorithm. **(D)** Violin plot depicting the expression of hub genes (ANPEP, MNDA, TLR7, FABP5, IL4I1, GM2A and C3) in different cell types.

To elucidate the expression patterns of genes associated with lipid metabolism within atherosclerotic plaques, we analyzed scRNA-seq data from the GSE159677 dataset. After quality control, we retained 43,465 high-quality cells for analysis. Through unsupervised clustering and dimensionality reduction, we identified eight principal cell lineages: B cells, endothelial cells, fibroblasts, macrophages, mast cells, natural killer (NK) cells, T cells, and vascular smooth muscle cells (VSMCs) (**Figures 5A, C**). The distribution of these cell types demonstrated significant variability across individual samples (**Figure 5B**). A bubble plot visualized the scaled expression levels and detection rates of canonical marker genes: NK cells (*NKG7, GNLY, KLRD1, KLRB1, PRF1*); fibroblasts (*DCN, LUM, IGF1, APOD, COL1A2*); mast cells (*VWA5A, SLC18A2, HDC*); endothelial cells (*RAMP2, EGFL7, RAMP3, PLVAP, VWF*); T cells (*CD3D, CD3E, CD8A, CD2*); B cells *(MS4A1, CD79B, VPREB3, BANK1, CD79A*); VSMCs (*CALD1, MYL9, MT1M, TAGLN*); and macrophages (*CD68, CD163, CD14*) (**Figure 5D**). A volcano plot highlighted genes with significant differential expression across the identified lineages (**Figure 6A**). Cell type-specific DEGs were identified using the FindAllMarkers function (Wilcoxon rank-sum test, **Supplementary Table S2**). Subsequent enrichment analysis of these DEGs revealed the top two significantly enriched pathways for each cell type (**Figure 6B**). Notably, macrophages exhibited a strong association with lysosomal pathway activity. To evaluate the activity of lipid metabolism-related genes at a single-cell level, we utilized the AUCell algorithm, which revealed that lipid-related gene activity was most pronounced in macrophages (**Figure 6C**). Violin plots depicted the expression distribution of the seven key LRGs across various cell types (**Figure 6D**). Our findings indicated that *ANPEP, MNDA*, and *GM2A* were predominantly expressed in macrophages, whereas the expression of the remaining four hub genes was largely absent in this population. Among these, *FABP5* was also expressed in NK cells, while *C3* was primarily observed in fibroblasts.

### 3.5 Identifies distinct macrophage subsets

**Figure 7:**
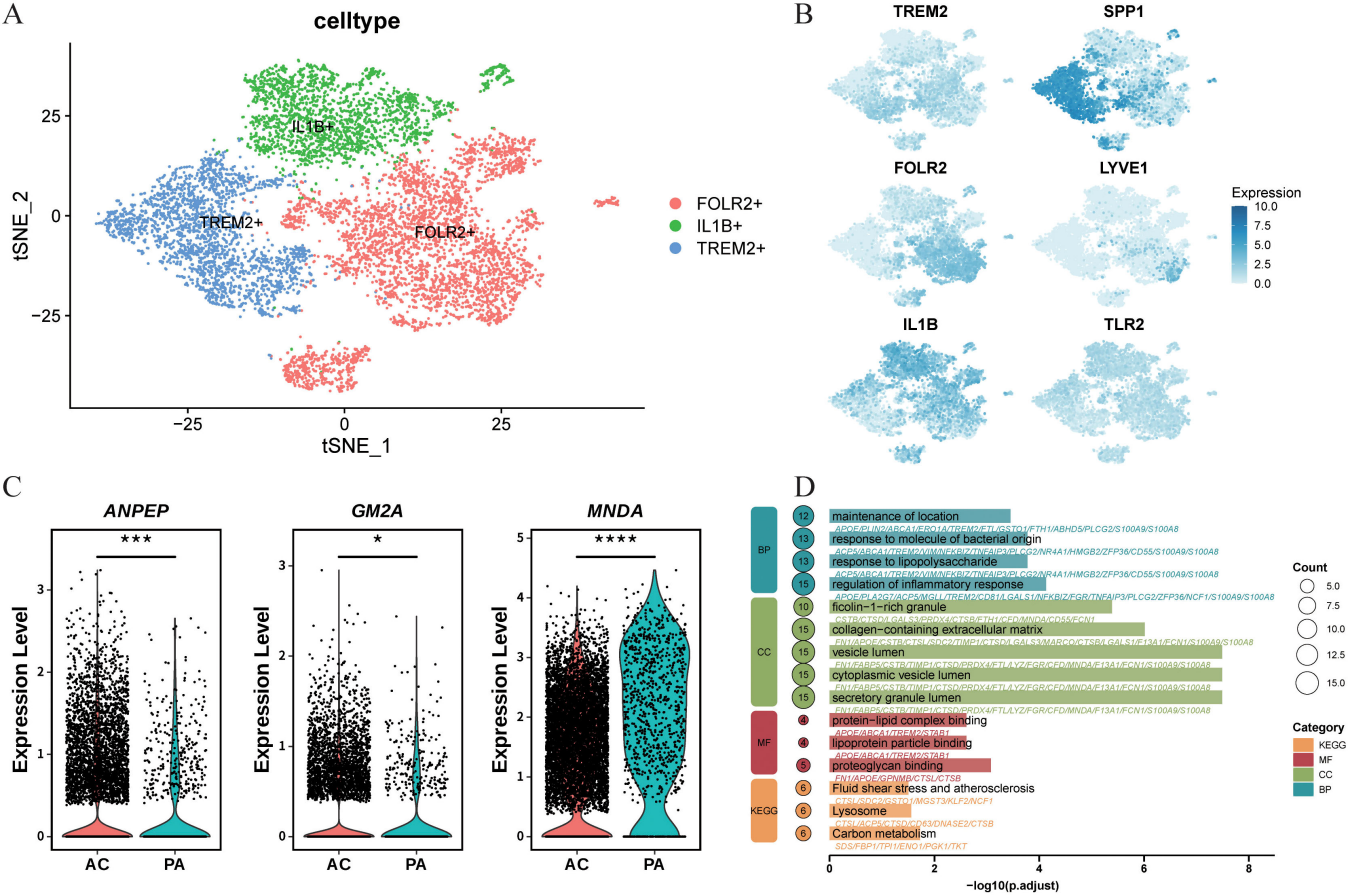
Distinct clusters of macrophage cells in atherosclerotic plaque. **(A)** Three macrophage atherosclerotic plaque cell subclusters were identified via UMAP analysis. **(B)** Feature plots for marker genes of macrophage cells. The color legend shows the expression levels of the genes. **(C)** Expression of three genes (ANPEP, MNDA and GM2A) in macrophages of atherosclerotic plaques across different groups **(D)** Functional enrichment analysis of macrophage cell-specific genes in atherosclerotic plaque, gene ontology (GO) analysis on the biological process (BP), cellular component **(CC)** and molecular function (MF). Kyoto Encyclopedia of genes and Genomes (KEGG) pathways. *P<0.05; ***P<0.001; ****P<0.0001.

Given that the hub genes (*ANPEP, MNDA*, and *GM2A*) are highly expressed in macrophages and that AUcell scores indicate the highest activity of LRGs in this lineage, we further dissected macrophage heterogeneity with single-cell data. Clustering resolved macrophages into three transcriptionally distinct subpopulations: FOLR2^+^, TREM2^+^, and IL1B^+,^each displaying a unique gene-expression signature (**Figure 7A**). Based on UMAP plots and violin plots of six genes (*TREM2, SPP1, FOLR2, LYVE1, IL1B, TLR2*) (**Figure 7B**; **Supplementary Figure S1**), we confirmed that macrophages comprise three distinct cell clusters (31). Among them, FOLR2^+^ macrophages are generally identified as tissue-resident macrophages involved in maintaining homeostasis and immunoregulatory functions, contrasting functionally with the TREM2^+^ subset (32). TREM2^+^ macrophages, frequently observed in various cancers and disease contexts, are termed lipid-associated macrophages and are closely associated with metabolic reprogramming (33, 34). IL1B^+^ macrophages represent a classical pro-inflammatory or M1-like phenotype, characterized by abundant production of pro-inflammatory cytokines (35, 36). Notably, *ANPEP*, *MNDA*, and *GM2A* were all markedly enriched in the AC group (**Figure 7D**).

### 3.6 Pseudotime trajectory analysis and cell communication analysis

**Figure 8:**
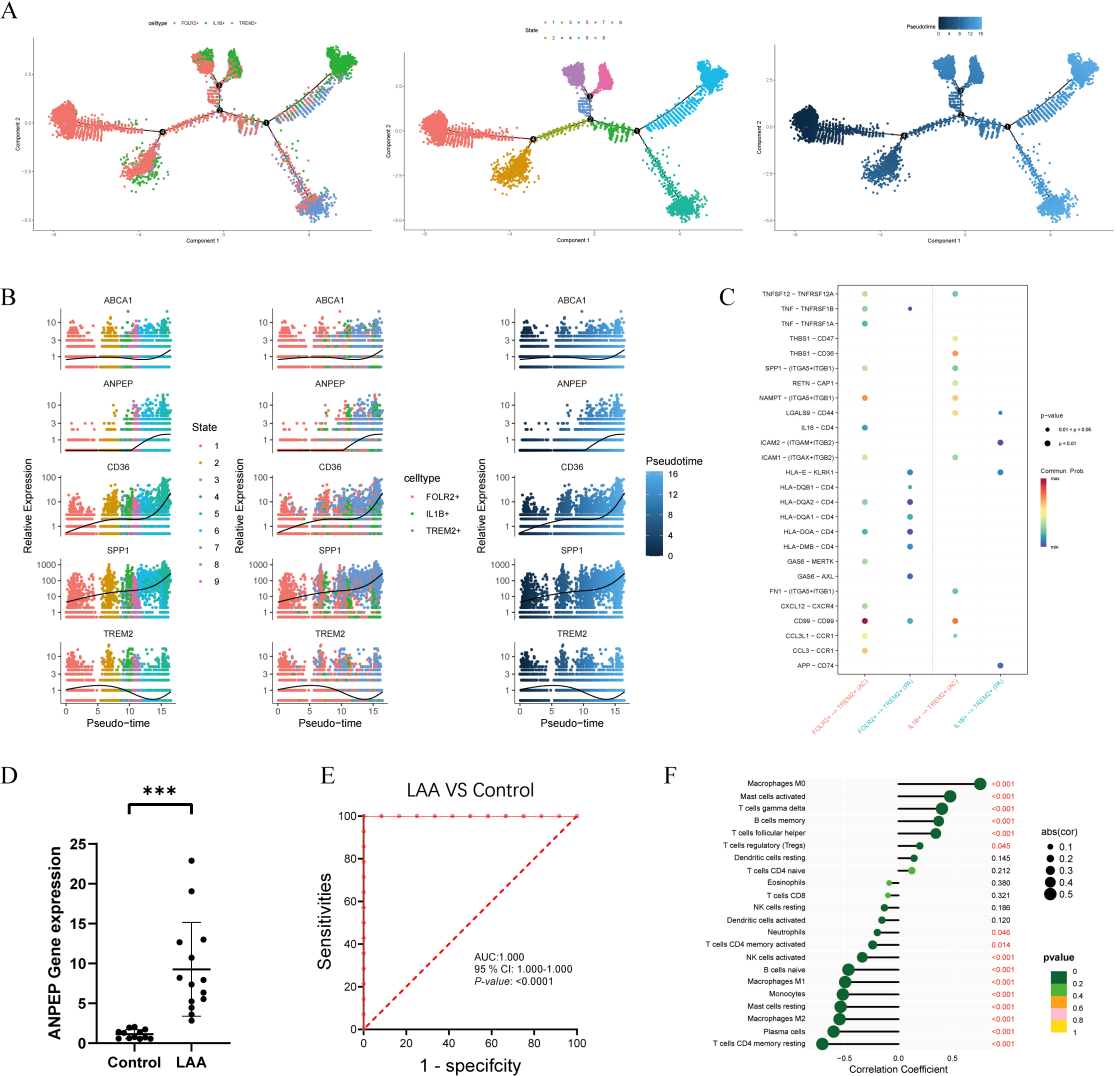
Analysis of the cellular developmental course and complex intercellular communication networks in atherosclerotic plaque, especially macrophage cells. **(A)** The pseudo temporal curve and its development trajectory of the cell types are depicted in the pseudo temporal trajectory diagram of monocle 2. The development pathway of each cell type and the differentiation mechanism of cell types as inferred by monocle 2. **(B)** Expression trend of key genes in macrophages with differentiation time. **(C)** Comparison of selective interactions mediated by ligand-receptor pairs between FOLR2+ and TREM2+ and IL1B+ and TREM2 +. Dot color reflects communication probabilities and dot size represents computed p-values computed from the one-sided permutation test. Empty space means the communication probability is zero. **(D)** The expression levels of ANPEP were analyzed by RT–qRT–PCR(A). **(E)** ROC curves of ANPEP. **(F)** Immune correlation lollipop chart of ANPEP in the GSE100927.

To investigate the differentiation pathways of macrophage subtypes within atherosclerotic plaques, Monocle 2 was utilized to analyze their trajectories. The findings indicated that FOLR2^+^ cells were in the initial stages of development (States 1 and 2), IL1B^+^ cells were in a transitional phase (States 3, 4, 7, 8, and 9), and TREM2^+^ cells had reached more terminal stage (States 5 and 6) (**Figure 8A**). Pseudotime analysis of *TREM2, SPP1, ANPEP, CD36*, and *ABCA1* demonstrated that the expression trajectory of *ANPEP* exhibited a strong correlation with those of *SPP1, CD36*, and *ABCA1* (**Figure 8B**), suggesting its potential pivotal role in lipid transport. Conversely, pseudotime analysis of other candidate genes (*MNDA*, *GM2A*) did not reveal consistent expression patterns (**Supplementary Figure S2**). Furthermore, the ‘CellChat’ algorithm was employed to assess intercellular signaling among macrophage subtypes (**Figure 8C**). The analysis revealed a complex intercellular communication network among all cell types(FOLR2+, TREM2+, and IL1B+ cells) present in the macrophages of atherosclerotic plaques. Numerous ligand-receptor pairs were identified, suggesting potential interactions between distinct subtypes. FOLR2^+^ and IL1B^+^ macrophages appeared to modulate TREM2^+^ cells via TNFSF12, SPP1, ICAM1 and CCL3L1, with the strongest influence observed within the AC group. These results highlight that TREM2^+^ cells may be regulated by FOLR2^+^ and IL1B^+^ macrophages in macrophages in advanced plaques.

To further validate *ANPEP* expression, we performed qRT–PCR analysis on clinically collected blood samples from 26 individuals (14 with LAA stroke and 12 healthy controls). The results demonstrated that *ANPEP* expression was significantly upregulated in the LAA stroke group compared to healthy controls (*p* < 0.001, **Figure 8D**). ROC analysis revealed that *ANPEP* achieved an AUC of 1.000 (95% CI: 1.000–1.000; *p* < 0.0001) in discriminating LAA stroke patients from healthy controls (**Figure 8E**), indicating excellent diagnostic performance in this cohort. To explore the potential role of *ANPEP* in immune regulation, we conducted Spearman correlation analysis to assess its relationship with immune cell infiltration in the GSE100927 dataset. *ANPEP* showed significant positive correlations with Macrophages M0, activated Mast cells, gamma delta T cells, memory B cells, follicular helper T cells, and regulatory T cells. Conversely, it was negatively correlated with Neutrophils, resting CD4 memory T cells, activated NK cells, naive B cells, Macrophages M1, Monocytes, resting Mast cells, Macrophages M2, Plasma cells, and activated CD4 memory T cells (**Figure 8F**). These findings collectively support ANPEP as a promising candidate biomarker for LAA stroke and suggest its involvement in modulating immune cell infiltration.

### 3.7 Integrated validation of ANPEP’s role in cellular function and lipid metabolism

**Figure 9:**
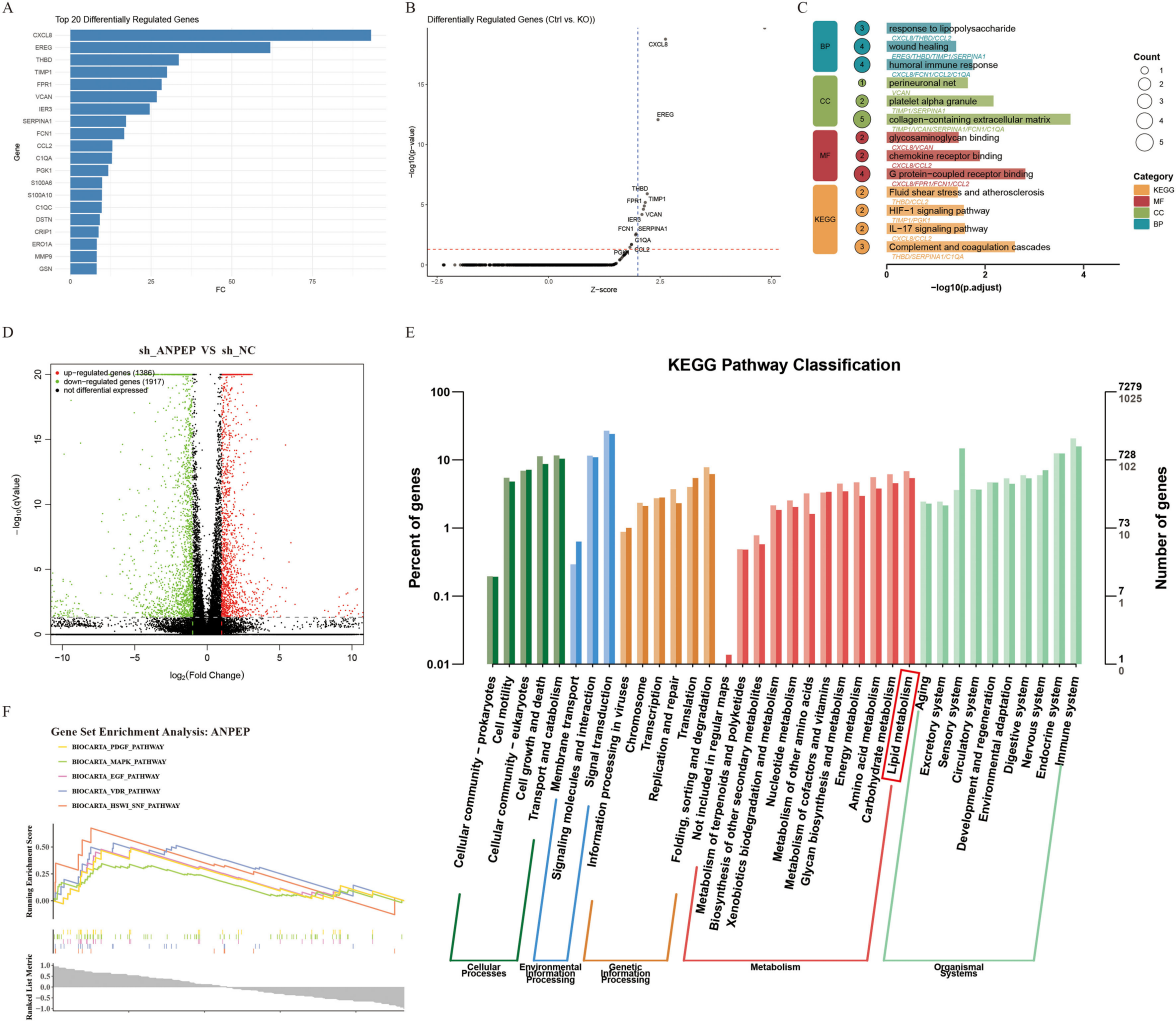
In-silico functional perturbation analysis of ANPEP knockout and post-knockout RNA-sequencing results. Knockdown of ANPEP can affect the lipid transport. **(A, B)** In-silico knockout of ANPEP in macrophages from atherosclerotic plaques identifies key differentially affected genes. (A: bar plot of top 20 genes; B: scatter plot). **(C)** Functional enrichment of the perturbed genes using KEGG and GO, suggesting disrupted lipid metabolism following ANPEP depletion. RNA sequencing showed that 1917 genes were downregulated and 1386 genes were upregulated after ANPEP knockdown in RAW264.7 cell **(D)**. GSEA analysis showed the enrichment results after ANPEP knockdown (p <0.001) **(F)**. In RAW264.7 cell with ANPEP knockdown, comprehensive enrichment analyses were performed using Kyoto Encyclopedia of Genes and Genomes (KEGG) **(E)**.

Among the signature genes, *ANPEP* was prominently expressed in macrophages. To functionally characterize ANPEP, we performed an in silico knockout in macrophages derived from atherosclerotic plaques (**Figures 9A, B**; **Supplementary Table S3**). Subsequent analysis revealed that key differentially expressed genes were enriched in pathways including complement and coagulation cascades, IL-17 signaling, HIF-1 signaling, and fluid shear stress response, all pathways implicated in atherosclerosis progression. These computational predictions suggest *ANPEP* may contribute to hemodynamic adaptation and vascular inflammatory regulation. Furthermore, GO analysis indicated that *ANPEP* knockdown was computationally associated with impairment in molecular functions such as G protein-coupled receptor binding, chemokine receptor binding, and glycosaminoglycan binding (**Figure 9C**). These processes are closely associated with cellular signal transduction and immune cell recruitment, suggesting that ANPEP might modulate macrophage function via inflammatory signaling networks.

To experimentally elucidate the role of ANPEP in lipid metabolism, we conducted RNA sequencing in *ANPEP*-knockdown RAW264.7 cell, identifying 3,303 DEGs—1,386 upregulated and 1,917 downregulated (**Figure 9D**). GSEA demonstrated that *ANPEP* depletion significantly influenced multiple pathways related to cell proliferation, signal transduction, and lipid metabolism (**Figure 9F**). Notably, both the VDR and MAPK pathways, known regulators of cholesterol metabolism and lipid-related gene expression, were markedly affected.

KEGG pathway analysis further corroborated these observations, showing significant enrichment in pathways involved in lipid and energy metabolism, environmental sensing, and genetic information processing (**Figure 9E**). Comprehensive GO analysis revealed that *ANPEP* knockdown broadly disrupted biological processes including cellular and organismal processes, metabolic and immune responses, and signal transduction (**Supplementary Figure S3A**). These changes suggest that *ANPEP* is integral to maintaining cellular homeostasis, and its loss may provoke systemic metabolic disturbances, including dysregulated lipid metabolism. In parallel, KOG analysis highlighted ANPEP’s influence on functional categories spanning RNA processing, chromatin organization, energy metabolism, cell motility, signal transduction, and lipid transport (**Supplementary Figure S3B**). The pronounced enrichment of lipid transport processes underscores ANPEP’s central role in lipid homeostasis. Our integrative analysis establishes ANPEP as a key regulator of cellular lipid metabolism. Its knockdown perturbs a spectrum of lipid-related pathways and processes, likely contributing to metabolic dysfunction.

### 3.8 Interference with *ANPEP* expression suppresses macrophage-derived foam cell formation

**Figure 10:**
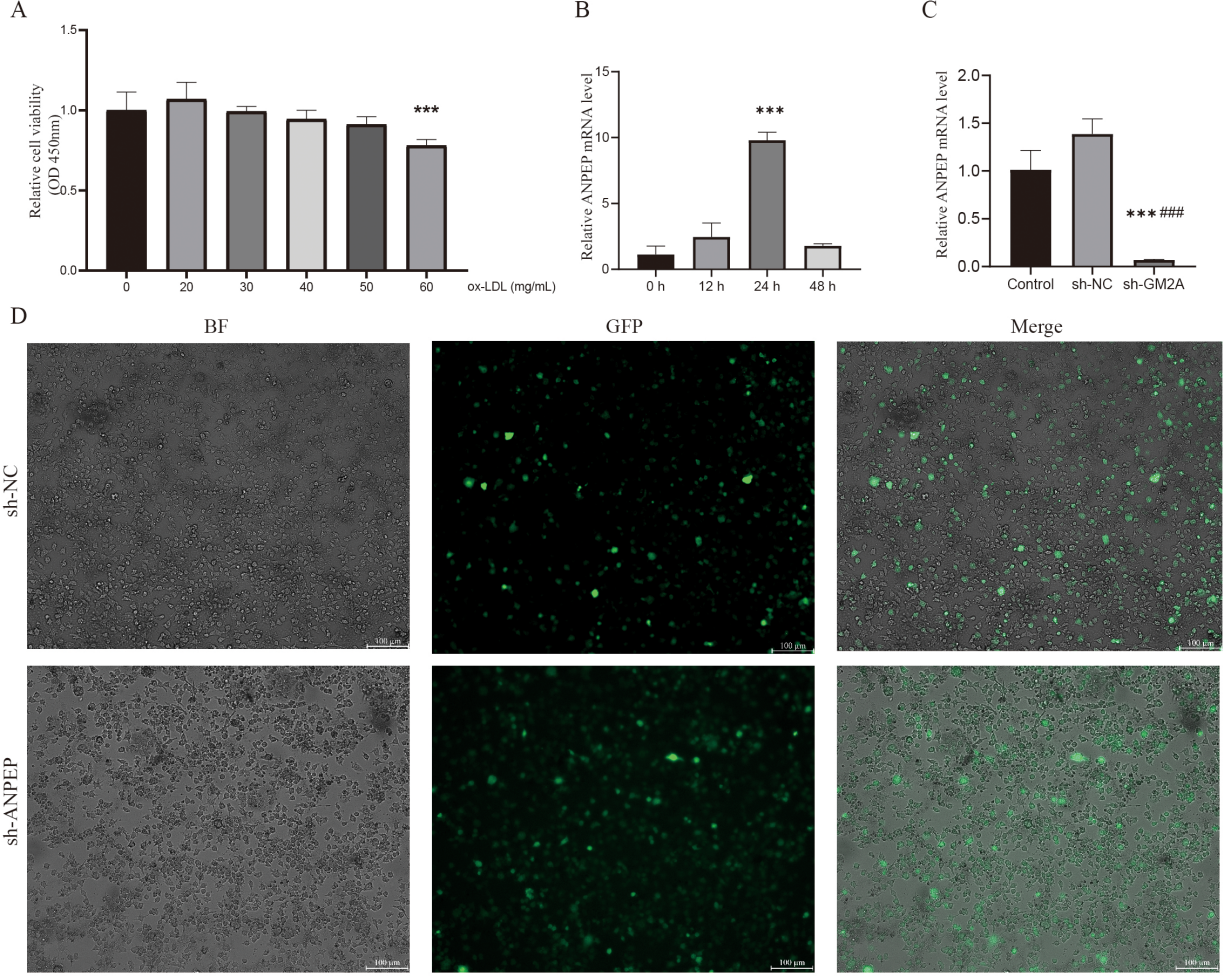
Foam cell model construction. Inhibition of the survival viability of RAW264.7 cell by ox-LDL, the RAW264.7 cell were treated with ox-LDL for 24 h and cell viability was measured by CCK 8 assay **(A)**. (The RAW264.7 cell were treated with ox-LDL at different concentrations 0,20,30,40,50,60 mg/mL for 24 h; ***, P<0.001, vs control). The RAW264.7 cell were treated with 50 mg/mL ox-LDLL for 12,24, and 48 h, and the intracellular ANPEP expression changes were measured by qRT-PCR **(B)**. (***, P<0.001, vs 0 h) After RAW 264.7 cells were transfected with plvx-shRNA2-ZSGreen-T2A-puro lentiviral vector and selected 14 days after 5 μg/mL puromycin selection **(D)**, the expression of sh-NC and sh-ANPEP in ANPEP was determined by qRT‒PCRplvx-shRNA2-ZSGreen-T2A-puro lentiviral vector **(C)**. (***, P<0.001 vs control; ###, P<0.001, vs sh-NC).

Ox-LDL induces the formation of macrophage-derived foam cells and is a major risk factor for atherosclerosis. To investigate the effect of ox-LDL on the proliferation of RAW264.7 cells, a CCK-8 assay was performed. Treatment with 50 μg/mL ox-LDL exhibited no obvious cytotoxicity, whereas cell viability was significantly decreased at 60 μg/mL, indicating a concentration-dependent cytotoxic effect (**Figure 10A**). Accordingly, 50 μg/mL ox-LDL was chosen to induce foam cell formation. Subsequent time-course analysis using qRT-PCR showed that ANPEP expression peaked at 24 h after ox-LDL stimulation, with lower levels detected at 12 h and 48 h (**Figure 10B**). Such temporal upregulation of ANPEP during foam cell formation suggests its potential regulatory role in this process. Therefore, stimulation with 50 μg/mL ox-LDL for 24 h was used in all subsequent experiments.

**Figure 11:**
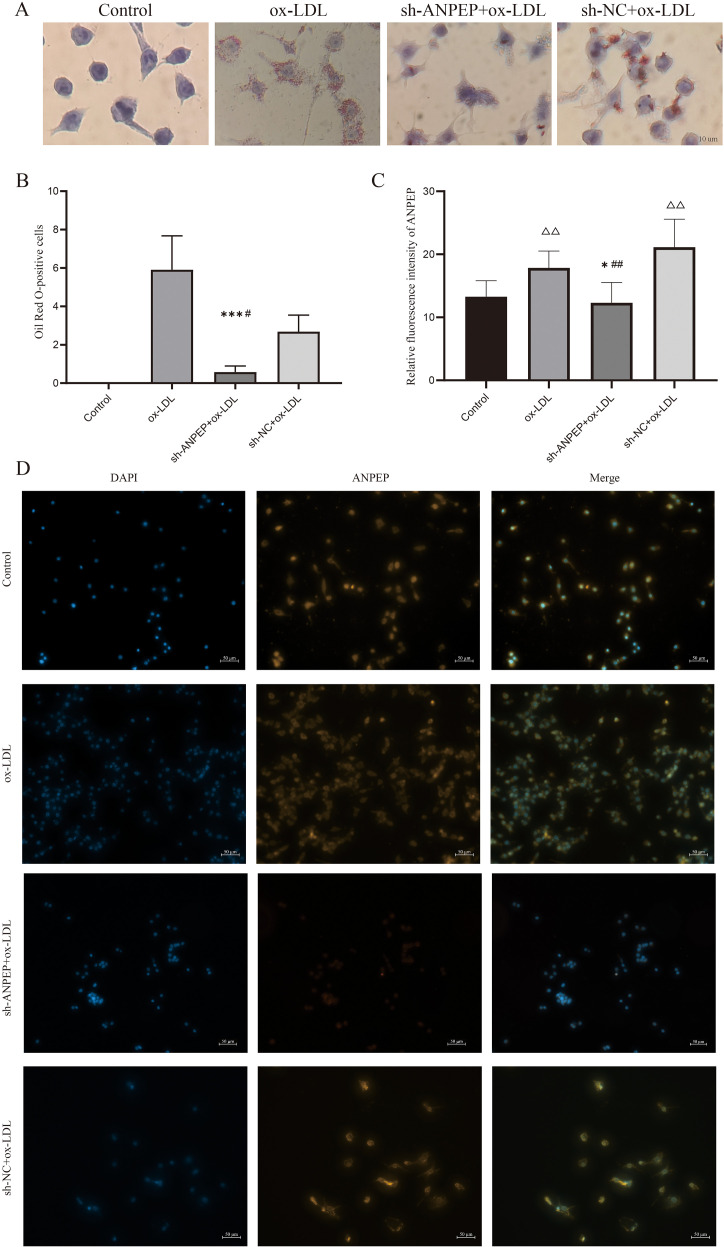
ANPEP expression in the foam cells. Intracellular lipid droplets were detected by oil red O staining **(A)**, and statistics of oil red O stained cells were made by Image J software **(B)**. The fluorescence. The expression of ANPEP in the cells was observed by fluorescence microscopy **(D)** and statistical analyzed by Image J software **(C)**. (△△, P<0.01, vs control; *, P<0.05, ***, P<0.001 vs ox-LDL; #, P<0.05, ##, P<0.01, vs sh-NC+ox-LDL).

To further confirm the role of ANPEP in ox-LDL-induced macrophage foam cell formation, we constructed a stable ANPEP knockdown macrophage cell line (sh-*ANPEP*) and a control cell line (sh-NC). Fluorescence microscopy showed approximately 80% infection efficiency (**Figure 10C**), and qRT-PCR confirmed significantly decreased ANPEP expression in sh-ANPEP cells compared to sh-NC cells (**Figure 10D**), indicating successful knockdown. During AS, macrophages convert into foam cells by phagocytosing excess lipids, accompanied by massive lipid droplet formation (37). To investigate whether ANPEP knockdown affects foam cell formation, we performed Oil Red O staining to visualize intracellular lipid droplets. As shown in **Figures 11A, B**, lipid droplets were significantly increased in the ox-LDL-treated group compared to the control group (*p* < 0.01), confirming successful foam cell induction. This increase was markedly attenuated upon ANPEP knockdown (sh-ANPEP + ox-LDL group vs. sh-NC + ox-LDL group, *p* < 0.05), demonstrating that inhibition of ANPEP expression could effectively reduce ox-LDL-induced lipid deposition and subsequent foam cell formation. To further elucidate the relationship between ANPEP expression and lipid accumulation, we examined intracellular ANPEP levels under the same conditions. Immunofluorescence staining revealed significantly increased intracellular ANPEP in the ox-LDL-treated group compared with the control group, suggesting that ANPEP may be upregulated in response to lipid loading. Conversely, intracellular ANPEP expression was significantly decreased in the sh-ANPEP + ox-LDL group compared with the sh-NC + ox-LDL group, confirming the efficiency of ANPEP knockdown. Taken together, these results demonstrate that ANPEP interfering lentivirus effectively downregulates ANPEP content in macrophages, and disruption of ANPEP expression attenuates ox-LDL-induced intracellular lipid deposition, highlighting ANPEP as a potential therapeutic target for mitigating foam cell formation in atherosclerosis (**Figures 11C, D**).

## 4 Discussion

Atherosclerosis is a primary pathogenic mechanism of cardiovascular disease and a major factor leading to ischemic stroke (38). The advent of multi-omics technology has profoundly advanced our understanding of the molecular mechanisms of atherosclerosis, leading to progress in clinical diagnosis and treatment strategies (39–41). In this study, we performed an integrative bioinformatics analysis based on the public transcriptome dataset GSE100927 and identified 441 differentially expressed genes (DEGs) between atherosclerotic patients and control subjects, including 120 upregulated and 321 downregulated genes. Functional enrichment analysis of overlapping DEGs revealed enrichment in terms related to immune cell involvement, immune activation, and inflammatory signaling, aligning with the established view that atherosclerosis is tightly associated with immunity and inflammation. We employed two machine-learning strategies, LASSO and SVM-RFE, to screen for critical gene signatures of atherosclerosis. Integrating multiple algorithms and taking the intersection of their results enhances the specificity of the identified markers. Ultimately, *ANPEP*, *MNDA*, *TLR7*, *FABP5*, *IL4I1*, *C3*, and *GM2A* were screened as characteristic gene signatures. ROC analysis demonstrated that elevated expression of these genes could accurately distinguish atherosclerosis from non-atherosclerotic individuals. Importantly, the expression of these genes maintained stable performance in external validation datasets. Our findings showed that *ANPEP*, *MNDA*, and *GM2A* were mainly expressed in macrophages, while the other four hub genes were mostly not present in these cells. FABP5 was also found in natural killer (NK) cells, and *C3* was mainly seen in fibroblasts. Since the hub genes (*ANPEP*, *MNDA*, and *GM2A*) are highly expressed in macrophages and AUcell scores highlight the highest lysosome-related gene activity in this lineage, we further analyzed macrophage heterogeneity using single-cell data.

Macrophage foam formation marks the initial stage of atherosclerotic plaque development, with lysosomes playing crucial roles in its progression (42). Single-cell data revealed that the pseudotime trajectory of ANPEP aligns with those of CD36 and ABCA1, genes crucial for lipid uptake and efflux, suggesting a potential coordinated role in macrophage metabolic processes. Cell communication analyses showed that TREM2^+^ macrophages exhibit robust interactions with other macrophage subgroups. Given that TREM2 promotes lipid uptake and foam cell formation in atherosclerosis, these interactions highlight a potential role for ANPEP in foam cell formation, underscoring its importance in the pathogenesis of atherosclerosis.

In this study, we found that ANPEP demonstrated promising diagnostic properties in serum qRT–PCR analysis of a clinical cohort. Our immune infiltration analysis suggested a potential relationship between ANPEP and macrophage differentiation, showing positive correlation with macrophages M0 and negative association with macrophages M1 and M2. This may be related to the role of macrophages in engulfing lipids to become foam cells during atherosclerosis. In subsequent experiments, ANPEP knockdown in RAW264.7 cells significantly impaired their lipid uptake capacity. Sequencing data revealed that DEGs following ANPEP knockout were significantly enriched in lipid metabolism pathways. These results were further supported by analysis of atherosclerotic clinical blood samples. Collectively, these findings suggest that ANPEP may be potentially involved in the pathogenesis of atherosclerosis. Recent studies have revealed that the lysosome-related gene ANPEP plays an important role in the development of lipid metabolism and atherosclerosis. Notably, modulating the intestinal expression of *ANPEP* and improving the circulating cholesterol distribution can attenuate atherosclerosis (43). ANPEP expression levels are correlated with atherosclerosis risk. In a study on unstable atherosclerotic plaque-related stroke, smoking was found to upregulate *ANPEP* expression, potentially accelerating plaque instability and stroke occurrence, suggesting that changes in *ANPEP* expression may be related to plaque stability and disease risk (44). *ANPEP* inhibitors have shown potential in treating atherosclerosis. Research indicates that 27-hydroxycholesterol can increase insulin-regulated aminopeptidase activity and enhance ANPEP levels, which in turn impairs neuronal glucose uptake. Lowering 27-hydroxycholesterol or inhibiting ANPEP may be useful strategies for preventing metabolic changes, implying that ANPEP inhibitors may influence atherosclerosis by regulating related pathways (45).

We demonstrate that ANPEP is crucial for cholesterol transport and cellular balance. Cholesterol buildup in macrophages can lead to foam cell formation and atherosclerosis. Our study found that ANPEP is upregulated by ox-LDL, as seen in disease samples. RNA sequencing suggests that ANPEP upregulation may hinder lipid efflux, contributing to foam cell formation. ANPEP may influence lipid metabolism through VDR and MAPK pathways, enhancing lipid uptake via scavenger receptors like CD36 and reducing cholesterol efflux by inhibiting ABCA1/ABCG1 transporters. These actions collectively increase lipid accumulation. These observations, based on transcriptomic analysis, suggest a potential role for VDR/MAPK signaling in ANPEP-driven foam cell formation, but require biochemical and pharmacological validation. Multi-omics data confirm that ANPEP impacts foam cell formation and atherosclerosis by affecting lipid metabolism and macrophage activity. In conclusion, targeting ANPEP may represent a promising therapeutic strategy for atherosclerosis and stroke. While our RNA sequencing data following ANPEP knockdown validate our initial in silico predictions and support a functional role in lipid efflux, the reliance on RAW264.7 cells, the small clinical cohort, and the correlative nature of the initial computational analysis represent limitations of this work. Future studies are therefore needed to confirm these effects in primary macrophages and to validate the clinical significance in larger patient populations. Addressing these aspects will be key to translating the mechanistic insights gained herein into improved clinical outcomes for patients with cardiovascular disease.

This study employs an integrative approach, combining multi-omics data with experimental validation, to elucidate the role of the lysosome-related gene *ANPEP* as a pivotal regulator of macrophage lipid metabolism and foam cell formation. Our findings demonstrate that *ANPEP* is markedly overexpressed in patients with atherosclerotic stroke, highlighting its significant diagnostic potential. Furthermore, *ANPEP* is specifically enriched at the single-cell level in TREM2^+^ lipid-associated macrophages. Functional assays reveal that the inhibition of *ANPEP* markedly diminishes ox-LDL-induced intracellular lipid accumulation, a process potentially linked to the modulation of lipid metabolism pathways, including VDR and MAPK signaling. These results underscore the significance of *ANPEP* as a prospective biomarker and provide a theoretical foundation for its consideration as a novel therapeutic target in the intervention of atherosclerotic stroke.

## Data Availability

The original contributions presented in the study are included in the article/**Supplementary Material**. Further inquiries can be directed to the corresponding authors.

## Supplementary material

Supplementary Table S1 Differential genes of GSE100927.

Supplementary Table S2 Characteristic gene expression profiles of various cell types in the GSE159677 dataset.

Supplementary Table S3 In silico knock-out result.

Supplementary Table S4 The table of Primer sequence.

Supplementary Figure S1 UMAP **(A)** and violin plots **(B)** for the six genes (“TREM2”, “SPP1”, “FOLR2”, “LYVE1”, “IL1B”, “TLR2”) in macrophages.

Supplementary Figure S2 Pseudotime analysis of candidate genes.

Supplementary Figure S3 In RAW264.7 cell with ANPEP knockdown, comprehensive enrichment analyses were performed using Gene Ontology (GO) **(A)** and Clusters of Orthologous Groups (KOG) **(B)** to elucidate the biological impacts of ANPEP knockdown.
