# Supplementary material for: ANPEP governs macrophage lipid metabolism and macrophage foam cell formation in large-artery atherosclerotic stroke
Source: Front Immunol. 2026 Apr 24;17:1747321. doi: 10.3389/fimmu.2026.1747321 (PMC13152831; doi:10.3389/fimmu.2026.1747321)
Supplement: Supplementary Figure S1 — UMAP (A) and violin plots (B) for the six genes (“TREM2”, “SPP1”, “FOLR2”, “LYVE1”, “IL1B”, “TLR2”) in macrophages. [file Table2.docx]

**Supplementary Material 2**

**
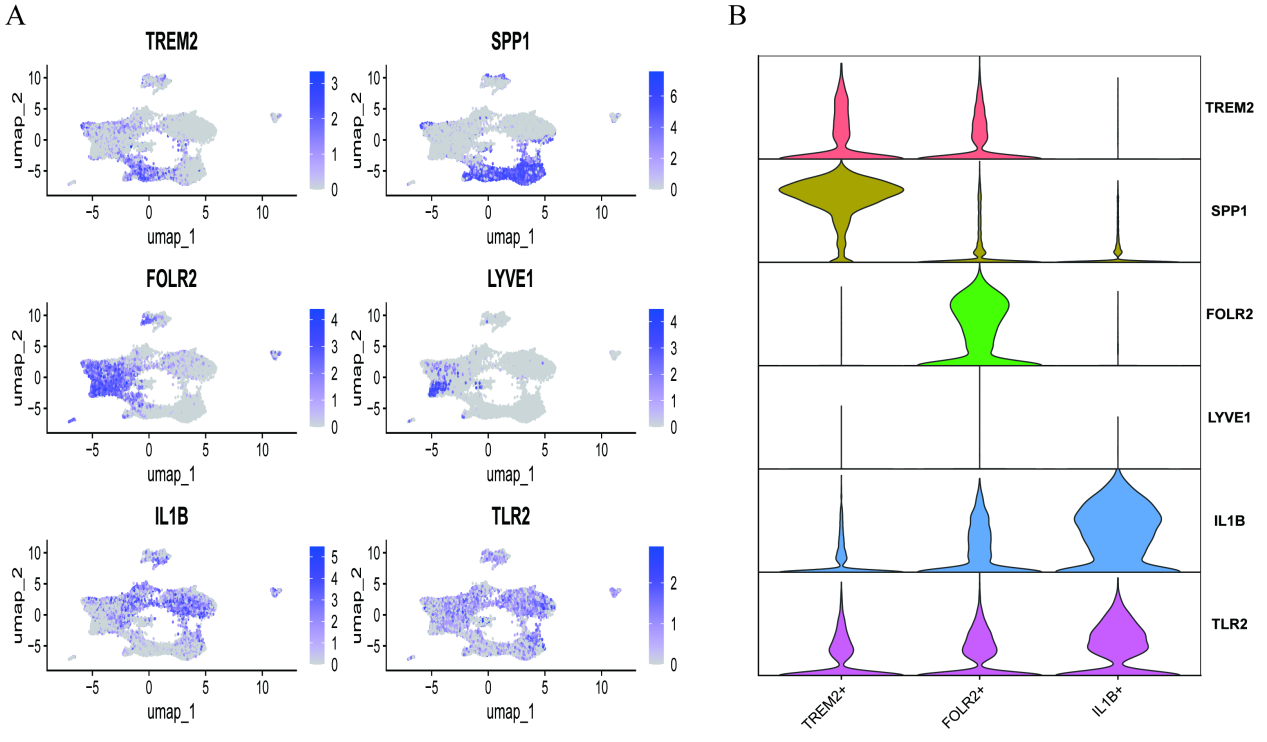
**

Figure S1: UMAP(A) and violin plots(B) for the six genes ("TREM2", "SPP1", "FOLR2", "LYVE1", "IL1B", "TLR2") in macrophages.


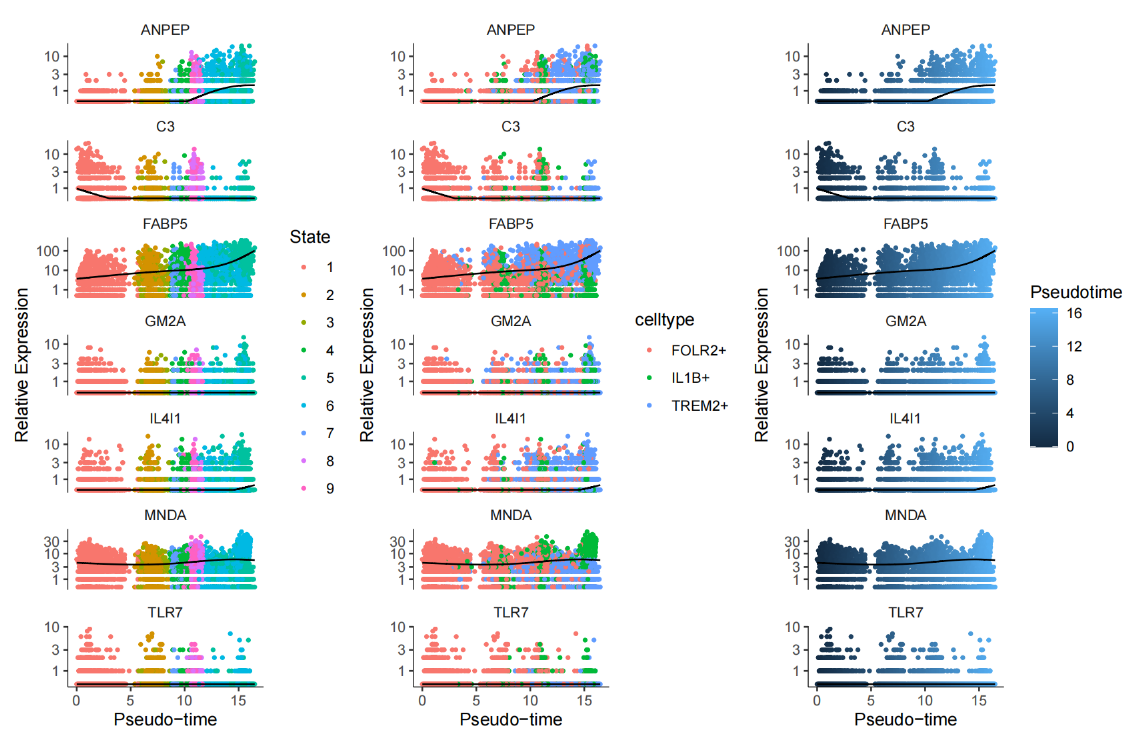


Figure S2: Pseudotime analysis of candidate genes

**
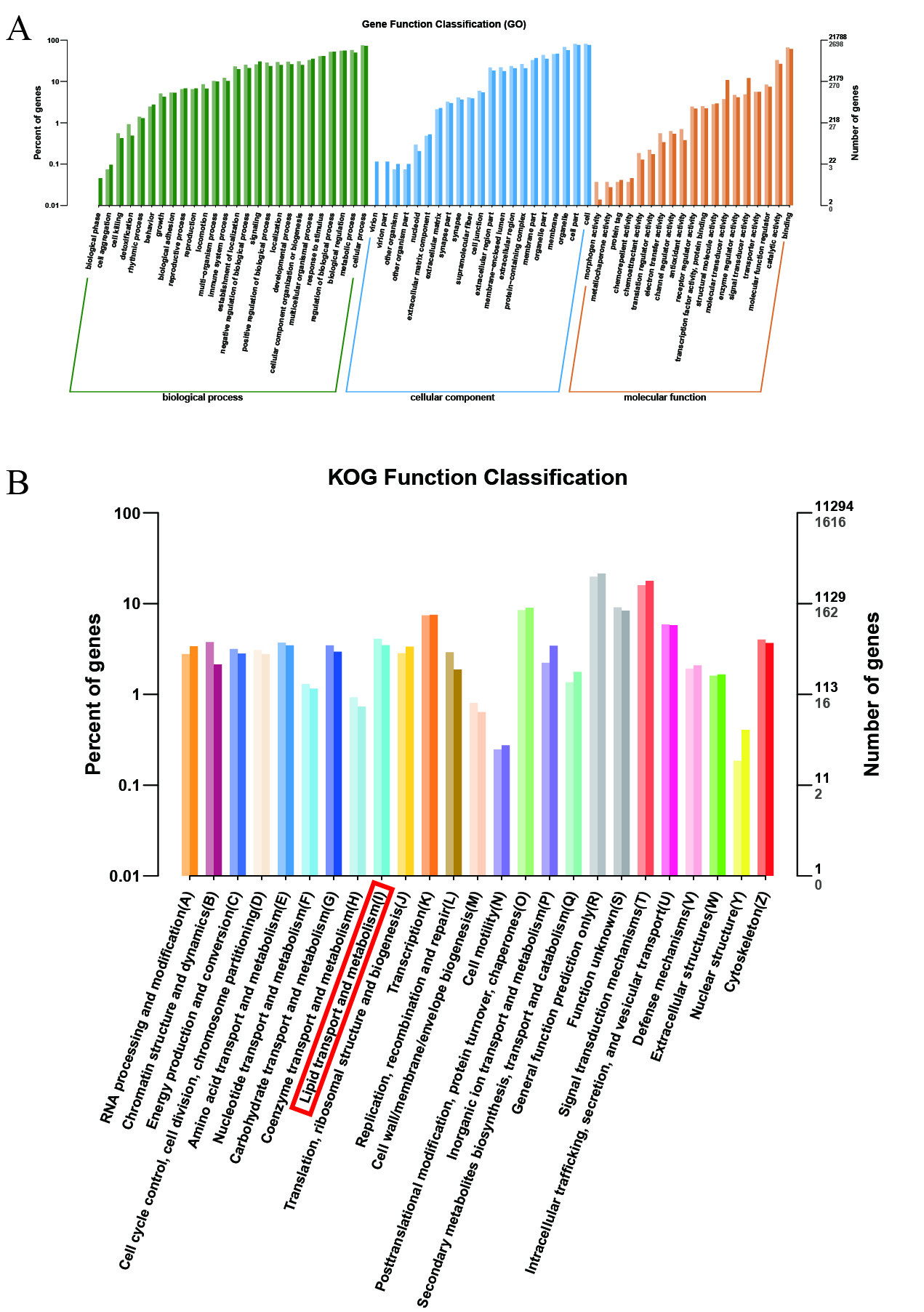
**

Figure S3: In RAW264.7 cell with ANPEP knockdown, comprehensive enrichment analyses were performed using Gene Ontology (GO)(A) and Clusters of Orthologous Groups (KOG)(B) to elucidate the biological impacts of ANPEP knockdown.
